# Detection of compound heterozygous variants in LPIN1 does not necessarily imply pathogenicity in a patient with rhabdomyolysis

**DOI:** 10.12688/f1000research.21589.1

**Published:** 2020-01-13

**Authors:** Josef Finsterer, Rahim Aliyev

**Affiliations:** 1 Neurological Department, Krankenanstalt Rudolfstiftung, Messerli Institute, Vienna, 1180, Austria; 2 Department of Neurology and Clinical Neurophysiology, Azerbaijan State Advanced Training Institute for Doctors named after A. Aliyev, Baku, Azerbaijan

**Keywords:** LPIN1, rhabdomyolysis, myoglobinurea, renal insufficiency, genetics

## Abstract

In a recent article by Yim *et al.*, a 15-month-old male is described who experienced severe rhabdomyolysis with a creatine-kinase value (CKV) of 127494 U/l one day after intramuscular injection of an unidentified drug by the general practitioner. Rhabdomyolysis was not attributed to this injected drug but to compound heterozygous variants in LPIN1. The study has a number of shortcomings. Triggers of rhabdomyolysis should be unequivocally identified, a more extensive family history should be taken, and previous CKVs should be provided. Functional and biochemical tests should be carried out to confirm or exclude pathogenicity of the LPIN1 variants.

## Correspondence

In a recent article, Yim *et al.* reported a 15-month-old male who experienced severe rhabdomyolysis with a creatine-kinase value (CKV) of 127494 U/l one day after intramuscular injection of an unidentified drug by the general practitioner (GP) ^1^. Rhabdomyolysis was not attributed to this injection but to compound heterozygous variants in *LPIN1* ^1^. Each of the parents carried one of these variants but was clinically unaffected ^1^. This correspondence article provides reasonings as to why the detection of heterozygous variants in *LPIN1* does not necessarily imply pathogenicity in this case.

Rhabdomyolysis may not only occur in mitochondrial disorders (beta-oxidation defects are mitochondrial disorders) and glycogenoses, but more frequently in response to drugs or toxins ^2^. Thus, it is crucial to find out which drug the GP injected one day prior to admission, not only to identify the compound, but also to ensure that other patients were not exposed to any hazardous risks due to a possibly toxic drug.

Since the variant c.1949_1967dupGTGTCACCACGCAGTACCA was classified as likely pathogenic, the variant c.2410G>C as a variant of unknown significance, and since one parent each carried one of either variants, it is conceivable that the parent who carried the variant c.1949_1967dupGTGTCACCACGCAGTACCA also had experienced muscle manifestations previously. However, the family history is described as negative for rhabdomyolysis, malignant hyperthermia, or neuromuscular disorders making the pathogenicity of this variant quite unlikely. However, an extended family history should also be taken from the grandparents from the mother’s and father’s side to assess if they ever experienced any muscle symptoms.

Since *LPIN1* variants have been previously reported in association with recurrent rhabdomyolysis ^3, 4^, it is quite likely that the index patient or one of the parents had elevated CKVs. Thus, the records of the index patient or the parent who carried the likely pathogenic *LPIN1* variant should be checked to see if these individuals ever showed elevated CKVs. Of particular interest are CKVs after birth, exercise, infection, anaesthesia, or application of drugs. This is because CKV may particularly increase with these conditions. Since the younger sister of the index patient also carried the compound heterozygous *LPIN1* variants, we should be informed about the course of the mother’s pregnancy with this younger child, about the sister’s CKVs at birth and later, and further follow-up, including genetic counselling.

In the case report ^1^, the patient has a second episode of rhabdomyolysis at the age of six years. Current medication the index patient was taking at the time of this second episode should be examined, and also if the cause could have been triggered by exercise ^5^. A male of 6 years of age most likely is lively and usually highly physically active.

Overall, this interesting case report has some limitations, which should be addressed before attributing rhabdomyolysis to the *LPIN1* variants. Triggers of rhabdomyolysis should be unequivocally identified, a more extensive family history taken, and previous CKVs provided. Functional and biochemical tests should be carried out to confirm or exclude pathogenicity attributed to *LPIN1* variants.

## Data availability

No data is associated with this article.
