# Supplementary material for: Detection of compound heterozygous variants in LPIN1 does not necessarily imply pathogenicity in a patient with rhabdomyolysis
Source: F1000Res. 2020 Jan 13;9:15. [Version 1] doi: 10.12688/f1000research.21589.1 (PMC7429921; doi:10.12688/f1000research.21589.1)
Supplement: Peer review history

# Peer review: Reviewer response for version 1

**DOI:** 10.5256/f1000research.23792.r68521

**Published:** 2020-08-28

**Authors:** Chiara Pizzamiglio

**Affiliations:** 1 MRC Centre for Neuromuscular Diseases, UCL Institute of Neurology and National Hospital for Neurology and Neurosurgery, Queen Square, London, UK

I have read with interest the correspondence from Josef Finsterer and Rahim Aliyev entitled “Detection of compound heterozygous variants in LPIN1 does not necessarily imply pathogenicity in a patient with rhabdomyolysis”.

 I agree that in the article by Yim *et al.* the triggers of rhabdomyolysis have not been clearly determined and discussed. More importantly, the drug that was injected before the first episode of rhabdomyolysis was not specified so it is not possible to exclude that it triggered the episode. The identification of the trigger is crucial as it can guide the process of the differential diagnosis (Quinlivan R and Jungbluth H, 2012). ^1^ 

 Yim *et al*. do not clearly state what type of genetic testing was performed in the patient, i.e. rhabdomyolysis panel, exome, or genome sequencing. This is important to understand what other genetic causes of recurrent rhabdomyolysis have been excluded in the patient, especially given the fact that a muscle biopsy was not performed.

 In heterozygous carriers, LPIN1 mutation can cause cramps, myalgia and can trigger statin-induced myotoxicity, although it is not uncommon for parents to be asymptomatic. However, in the text, it is not specified if parents were taking statin.

 Despite the points discussed by Finsterer and Aliyev that should be addressed in the original article, the recurrent rhabdomyolysis, the fever in both episodes, the age of onset, the normal CK and examination between episodes, are in line with descriptions of LPIN1 associated rhabdomyolysis.

Are arguments sufficiently supported by evidence from the published literature or by new data and results?

Yes

Is the conclusion balanced and justified on the basis of the presented arguments?

Yes

Is the rationale for commenting on the previous publication clearly described?

Yes

Are any opinions stated well-argued, clear and cogent?

Yes

Reviewer Expertise:

Inherited muscular disorders, mitochondrial diseases, rhabdomyolysis.

I confirm that I have read this submission and believe that I have an appropriate level of expertise to confirm that it is of an acceptable scientific standard.

## References

[1] : Myopathic causes of exercise intolerance with rhabdomyolysis. *Dev Med Child Neurol*.2012;54(10) : 10.1111/j.1469-8749.2012.04320.x 886-91 10.1111/j.1469-8749.2012.04320.x 22616958

# Peer review: Reviewer response for version 1

**DOI:** 10.5256/f1000research.23792.r68527

**Published:** 2020-08-17

**Authors:** Pushpa Raj Joshi

**Affiliations:** 1 Department of Neurology, Martin-Luther-University Halle-Wittenberg, Halle, Germany

The correspondence ‘Detection of compound heterozygous variants in LPIN1 does not necessarily imply pathogenicity in a patient with rhabdomyolysis’ by Josef Finsterer and Rahim Aliyev well argue against the conclusion of original submission ‘Case Report: The first probable Hong Kong Chinese case of LPIN1-related acute recurrent rhabdomyolysis in a boy with two novel variants’ by SW Yim and colleagues. In this submission, the authors are not sure which drug was administered to the patient prior to admission and Dr. Finisterer and Dr. Aliyev rightly point out that Rhabdomyolysis should not have necessarily be triggered by the LPIN1 mutations but might have been due to the effect of the compounds in the injection. SW Yim and colleagues should find out the composition of the injection and prove that rhabdomyolysis was not triggered by any of the compounds in the injection.

 On the other hand, it is very likely that the conclusion of SW Yim might be correct. Although the pathogenicity of the reported mutations is not clear, the compound heterozygosity might be the cause of rhabdomyolysis and the mutations are, in fact, pathogenic. This is strongly supported by the fact that the child suffered from the second attack of rhabdomyolysis. However, the report on detailed family history, as pointed out by Dr. Finisterer and Dr. Aliyev, will be helpful in arguments about the pathogenicity of the mutations. Despite autosomal recessive inheritance of LPIN1 mutations, the parent(s) with only one mutation might also suffer from attack(s) of rhabdomyolysis with very mild to severe intensity. Symptomatic heterozygous cases are sporadically reported in other autosomal recessive disorders such as CPT II deficiency, also contributing to rhabdomyolysis.

 Overall, the original submission is interesting but needs to address the points raised by Dr. Finisterer and Dr. Aliyev to draw the conclusion that rhabdomyolysis is triggered by novel LPIN1 mutations.

Are arguments sufficiently supported by evidence from the published literature or by new data and results?

Yes

Is the conclusion balanced and justified on the basis of the presented arguments?

Yes

Is the rationale for commenting on the previous publication clearly described?

Yes

Are any opinions stated well-argued, clear and cogent?

Yes

Reviewer Expertise:

Meuromuscular disordres, mitochondrial myopathy, Rhabdomyolysis

I confirm that I have read this submission and believe that I have an appropriate level of expertise to confirm that it is of an acceptable scientific standard.

# Peer review: Reviewer response for version 1

**DOI:** 10.5256/f1000research.23792.r68525

**Published:** 2020-08-13

**Authors:** Karolina M. Stepien

**Affiliations:** 1 Mark Holland Metabolic Unit, Adult Inherited Metabolic Diseases Department, Salford Royal NHS Foundation Trust, Salford, UK

I note the comments from Finsterer and Aliyev.

 I agree that it would be worth learning more about the first episode of rhabdomyolysis in this child who was confirmed to be compound heterozygous for two mutations causing LIPIN1. A causal relationship between this episode and the administered injection needs to be considered, and detail is given on what the injection was including dose, route, number of doses, indication, etc. This is a fundamental point that has not been addressed in the case report. Drug-induced rhabdomyolysis is relatively common.

 Although 40% of heterozygous carriers for LPIN1 missense mutations may be symptomatic, myopathy has been reported in these individuals in response to statin drug treatment (Zhang *et al. *2014) ^1^.

 The authors listed several factors that may trigger rhabdomyolysis in LIPIN1. The fact that in humans episodes of myoglobinuria in LIPIN1 are mostly precipitated by febrile illnesses (Michot *et al.* 2014) ^2^ emphasizes a critical role of the inflammatory stress response as a potential triggering factor of rhabdomyolysis.

 I agree with the authors that there are some limitations of the case report by Yim *et al. *2019. Firstly, a CK of 127,000 U/L may have detrimental consequences in a toddler. Dexamethasone was shown to decrease the number of lipid droplets in lipin‐1 deficient patients' myoblasts with a decrease in peak CK concentration during acute rhabdomyolysis. This therapy may prove to be beneficial for severe decompensation (Maijer *et al. *2015) ^3^. Was it considered during the acute illness? What about renal function and fluid management?

Are arguments sufficiently supported by evidence from the published literature or by new data and results?

Yes

Is the conclusion balanced and justified on the basis of the presented arguments?

Yes

Is the rationale for commenting on the previous publication clearly described?

Yes

Are any opinions stated well-argued, clear and cogent?

Yes

Reviewer Expertise:

Inherited Metabolic Diseases

I confirm that I have read this submission and believe that I have an appropriate level of expertise to confirm that it is of an acceptable scientific standard.

## References

[1] : Lipin-1 regulates autophagy clearance and intersects with statin drug effects in skeletal muscle. *Cell Metab*.2014;20(2) : 10.1016/j.cmet.2014.05.003 267-79 10.1016/j.cmet.2014.05.003 24930972PMC4170588

[2] : LPIN1 gene mutations: a major cause of severe rhabdomyolysis in early childhood. *Hum Mutat*.2010;31(7) : 10.1002/humu.21282 E1564-73 10.1002/humu.21282 20583302

[3] : LPIN1 deficiency with severe recurrent rhabdomyolysis and persistent elevation of creatine kinase levels due to chromosome 2 maternal isodisomy. *Mol Genet Metab Rep*.2015;5: 10.1016/j.ymgmr.2015.10.010 85-88 10.1016/j.ymgmr.2015.10.010 28649549PMC5471397

# Peer review: Reviewer response for version 1

**DOI:** 10.5256/f1000research.23792.r68520

**Published:** 2020-08-06

**Authors:** Panupong Hansrivijit

**Affiliations:** 1 Department of Internal Medicine, University of Pittsburgh Medical Center Pinnacle, Harrisburg, PA, USA

Thank you for pointing out other etiologies that the original authors might have missed. However, I agree with the original authors' conclusion that rhabdomyolysis in this infant is related to spontaneous rhabdomyolysis from compound heterozygous variants of LPIN1 but the fact that we do not know what medication was given has raised some concerns for the accuracy of the diagnosis.

 Please add another reference that covers the genetic perspectives of rhabdomyolysis as attached. ^1^ I believe this additional article will be helpful to the readers who are interested in reading up further.

Are arguments sufficiently supported by evidence from the published literature or by new data and results?

Yes

Is the conclusion balanced and justified on the basis of the presented arguments?

Yes

Is the rationale for commenting on the previous publication clearly described?

Yes

Are any opinions stated well-argued, clear and cogent?

Yes

Reviewer Expertise:

Minor revision is advised without the need to return the article to me.

I confirm that I have read this submission and believe that I have an appropriate level of expertise to confirm that it is of an acceptable scientific standard.

## References

[1] : Rhabdomyolysis: a genetic perspective. *Orphanet J Rare Dis*.2015;10: 10.1186/s13023-015-0264-3 51 10.1186/s13023-015-0264-3 25929793PMC4522153
